# Draft genome sequence of *Aliihoeflea* sp. strain PC F10.4, isolated from the Antarctic red alga *Plocamium cartilagineum*

**DOI:** 10.1128/mra.00135-26

**Published:** 2026-03-30

**Authors:** Waldo Acevedo, Fuad Mobarec, Jenifer Alvarez, Edith González-Ramos, Francisco Morera, Luis Vargas-Chacoff, Sergio Leiva Poveda

**Affiliations:** 1 Institute of Chemistry, Faculty of Sciences, Pontificia Universidad Católica de Valparaíso 28047, Valparaíso, Chile; 2 Center for Interdisciplinary Research in Biomedicine, Biotechnology and Well-Being (CID3B), Pontificia Universidad Católica de Valparaíso 28047, Valparaíso, Chile; 3 Institute of Biochemistry and Microbiology, Faculty of Sciences, Universidad Austral de Chile 28040, Valdivia, Chile; 4 School of Veterinary Medicine, Pontificia Universidad Católica de Chile 28033, Santiago, Chile; 5 Institute of Marine and Limnological Sciences, Faculty of Sciences, Universidad Austral de Chile 28040, Valdivia, Chile; 6 Instituto Milenio Biodiversidad de Ecosistemas Antárticos y Sub-Antárticos (BASE), Universidad Austral de Chile 28040, Valdivia, Chile; Montana State University, Bozeman, Montana, USA

**Keywords:** *Aliihoeflea*, Antarctica, macroalgae, *Plocamium cartilagineum*

## Abstract

We present the draft genome sequence of *Aliihoeflea* strain PC F10.4, an alphaproteobacterium isolated from the surface of the red macroalga *Plocamium cartilagineum* collected at King George Island, Antarctica. The assembled genome is 4,743,656 bp in length, with a G + C content of 61.8% and 4,628 protein-coding genes.

## ANNOUNCEMENT

*Aliihoeflea* is a little-studied, monotypic genus of gram-negative, rod-shaped alphaproteobacteria. Its only described species, *A. aestuarii* (1), was first isolated from tidal-flat sediments and designated as a new genus within the *Phyllobacteriaceae*. Most functional insight derives from *Aliihoeflea* sp. 2WW, studied for its arsenite-oxidizing capacity and bioremediation potential (2). A strain of *Aliihoeflea*, designated PC F10.4, was isolated from a healthy *Plocamium cartilagineum* specimen collected in the subtidal zone of Shoa Island (62°12′12″ S, 58°56′37″ W), King George Island, Antarctica. Its Antarctic origin and phylogeny highlight the value of sequencing this strain to explore the genus’s ecological and functional diversity.

Strain PC F10.4 was isolated from the surface of *P. cartilagineum* on marine agar 2216 (BD) at 20°C. Microbiological processing of macroalgal samples and isolation of pure cultures followed by Sánchez Hinojosa et al. (3). Genomic DNA was extracted from 72-h marine broth 2216 cultures at 20°C (100 rpm), using the GeneJET Genomic DNA Purification Kit and quantified with a Qubit fluorometer. Whole-genome sequencing was carried out at AUSTRAL–omics (Valdivia, Chile). Libraries were prepared from 400 ng of DNA using the Native Barcoding Kit 24 V14 (Oxford Nanopore Technologies [ONT]). Bead-based clean-up was performed according to the ONT protocol, without additional DNA shearing or size selection. Sequencing was performed on a MinION using a Flongle (R10.4.1) flow cell. Basecalling, adapter trimming, and quality filtering were performed with Dorado v0.5.1 (high-accuracy model; https://github.com/nanoporetech/dorado). Read quality was assessed with NanoPlot v1.42.0. *De novo* assembly was performed with Flye v2.9.1, and the resulting assemblies were polished with Medaka v2.0.0. ANI was calculated with FastANI (4) and dDDH with TYGS (5).

The complete 16S rRNA gene was retrieved from whole-genome data. EzBioCloud 16S database v.2025.04.21 (https://www.ezbiocloud.net/) (6) was used for sequence comparison. PC F10.4 exhibited 99.29% sequence similarity with *Aliihoeflea aestuarii* N8^T^ (EF660756) and 98.2% with *Aliihoeflea* sp. 2WW (HF565048). The ANI values between strain PC F10.4 and *Aliihoeflea aestuarii* JCM 15118^T^ (JAAAMK000000000) and *Aliihoeflea* sp. 2WW (AYOD00000000) are 84.67% and 82.71%, with corresponding dDDH (formula d4) values of 26.8% and 23.4%. These values are well below species thresholds, indicating that PC F10.4 is a putative novel *Aliihoeflea* species.

Oxford Nanopore sequencing produced 98,915 reads (635.8 Mb total) with a raw read *N*_50_ of 12,888 bp. PC F10.4 sequencing reads were assembled into five contigs, with an average coverage of 84.4× and an *N*_50_ of 4.4 Mb. The genome size is 4,743,656 bp, with 61.8% G + C content. It comprises 4,628 predicted protein-coding genes and 61 RNA genes (6 rRNA, 51 tRNA, and 4 ncRNA). According to NCBI’s CheckM-based analysis (v1.2.4) (7), assembly completeness is 97.02%.

Arsenic resistance genes *arsR*, *arsC*, *arsH*, and ACR3-family arsenite efflux transporter genes (8) were identified in the genome using NCBI PGAP v6.10 (9), and confirmed by BLASTp against characterized Ars proteins (≥70% amino-acid identity and ≥80% alignment coverage). This suggests a role of PC F10.4 in arsenic biogeochemical cycling in Antarctic environments and highlights its potential for the bioremediation of arsenic-contaminated waters. Given Chile’s mining- and metallurgical-related arsenic contamination (10), this strain may be a valuable resource for environmental protection.

## Data Availability

All data are available under BioProject number PRJNA1401851. Raw reads are deposited as accession number SRX31790216 and the genome assembly under GenBank accession number JBTSWH000000000. This is the first version of the genome.
